# Motor-Sparing Regional Analgesia in Frail and Sarcopenic Older Adults Undergoing Total Knee Arthroplasty: From Anatomy to Clinical Decision-Making—A Narrative Review

**DOI:** 10.3390/jcm15156071

**Published:** 2026-08-04

**Authors:** Paweł Pietraszek, Tomasz Reysner, Anna Kluzik, Anna Perek, Justyna Marszałek-Buko, Alicja Bartkowska-Śniatkowska, Katarzyna Wieczorowska-Tobis, Malgorzata Reysner

**Affiliations:** 1 Department of Clinical Anaesthesiology and Pain Management, Poznan University of Medical Sciences, 60-806 Poznan, Poland; anna.perek@ump.edu.pl (A.P.); justyna.marszalekbuko@ump.edu.pl (J.M.-B.); mreysner@ump.edu.pl (M.R.); 2 Department of Paediatric Anaesthesiology and Intensive Care, Poznan University of Medical Sciences, 60-806 Poznan, Poland; treysner@ump.edu.pl (T.R.); asniatko@ump.edu.pl (A.B.-Ś.); 3 Department of Anaesthesiology and Intensive Care, Poznan University of Medical Sciences, 60-806 Poznan, Poland; akluzik@ump.edu.pl; 4 Department of Palliative Medicine, Poznan University of Medical Sciences, 60-806 Poznan, Poland; kwt@tobis.pl

**Keywords:** total knee arthroplasty, motor-sparing analgesia, frailty, sarcopenia, regional anaesthesia, adductor canal block, mobility, functional independence, older adults, enhanced recovery after surgery

## Abstract

Total knee arthroplasty (TKA) is increasingly performed in older adults, a population with a growing prevalence of frailty and sarcopenia. Analgesic strategies focused mainly on pain reduction may overlook mobility, functional independence, fall prevention, and delirium risk, making motor preservation a central goal of perioperative care. This narrative review examines the anatomical basis, clinical evidence, and practical application of motor-sparing regional analgesia for older adults undergoing TKA, with emphasis on frailty, sarcopenia, and mobility-centred recovery, synthesising current evidence on established, emerging, and experimental techniques. Evidence was classified as geriatric-specific, older-enriched mixed-age, or mixed-age adult. Most technique-specific evidence derives from cohorts that included younger adults; only two small randomised trials reported cohorts composed exclusively of patients aged ≥65 years, and no regional-analgesia trial stratified outcomes by frailty or sarcopenia. A pragmatic clinical decision framework was developed to support individualised strategy selection in frail and sarcopenic patients. Current evidence supports adductor canal block combined with local infiltration analgesia as the foundation of motor-sparing analgesia after TKA; supplementary techniques such as iPACK, popliteal plexus block, genicular nerve block, and anterior femoral cutaneous nerve block may further optimise sensory coverage while preserving quadricep strength, whereas femoral nerve block produces predictable motor impairment and appears less suitable for this population. The existing literature remains largely focused on pain scores and opioid consumption, while mobility-related outcomes are infrequently reported. Direct evidence for falls, near-falls, delirium, discharge destination, functional independence, and long-term mobility remains sparse or absent. Successful analgesia after TKA should be defined not only by pain relief but by preservation of mobility and functional recovery, and future research should prioritise frailty-specific, mobility-centred outcomes.

## 1. Introduction

Total knee arthroplasty (TKA) is one of the most frequently performed orthopaedic procedures worldwide and remains the definitive treatment for end-stage knee osteoarthritis [1]. The demand for TKA continues to increase as populations age and life expectancy rises, with older adults representing the fastest-growing subgroup of patients undergoing knee replacement surgery [2]. Although TKA reliably improves pain, function, and quality of life, postoperative recovery remains highly dependent on the effectiveness of perioperative pain management [3].

Poorly controlled postoperative pain may limit participation in rehabilitation and overall functional recovery [4]. Traditionally, postoperative analgesia relied heavily on opioid administration. However, opioid-centred strategies are increasingly recognised as problematic, particularly in older adults [5]. Age-related physiological changes alter drug pharmacokinetics and pharmacodynamics, increasing susceptibility to respiratory depression, sedation, nausea, constipation, urinary retention, cognitive dysfunction, and postoperative delirium [6,7]. These complications may delay rehabilitation, prolong hospitalisation, and contribute to persistent functional decline [8].

At the same time, the demographic profile of patients undergoing TKA is changing. Increasing numbers of patients present not only with advanced age but also with frailty and sarcopenia [9]. Frailty reflects a state of diminished physiological reserve and increased vulnerability to stressors, whereas sarcopenia is characterised by progressive loss of skeletal muscle mass, strength, and physical performance [10,11]. Both conditions are independently associated with poorer postoperative outcomes, including prolonged recovery, increased risk of falls, non-home discharge, readmission, and mortality [12]. Pre-existing deficits in lower-limb muscle strength may substantially reduce these patients’ ability to compensate for additional postoperative motor impairment [13].

These observations challenge the traditional paradigm of evaluating regional anaesthesia techniques primarily according to pain scores and opioid consumption [14]. In younger and physiologically robust individuals, transient quadricep weakness may be clinically acceptable if accompanied by superior analgesia. In frail and sarcopenic older adults, however, even modest reductions in quadricep function may impair ambulation, delay rehabilitation, increase fall risk, and ultimately compromise the very outcomes that arthroplasty aims to improve [13,15]. Motor preservation is therefore increasingly viewed as an essential part of perioperative care [16].

Recent advances in regional anaesthesia have led to the development of several motor-sparing techniques designed to provide effective analgesia while minimising impairment of quadricep strength [14]. These include adductor canal block (ACB), femoral triangle block (FTB), local infiltration analgesia (LIA), genicular nerve block (GNB), infiltration between the popliteal artery and capsule of the knee (iPACK), popliteal plexus block (PPB), anterior femoral cutaneous nerve block (AFCNB), dual subsartorial block (DSB), triple-injection peri-sartorius block (TIPS), and erector spinae plane block (ESPB) [15,16,17,18,19,20]. Although several of these approaches have been evaluated in randomised controlled trials and meta-analyses, no regional-analgesia trial has been designed specifically for patients with frailty or sarcopenia. Consequently, most technique-specific recommendations for these groups are derived from mixed-age or physiologically selected adult cohorts and, where direct clinical evidence is absent, are supported by anatomical reasoning and geriatric principles.

The growing emphasis on Enhanced Recovery After Surgery (ERAS) pathways further reinforces the importance of motor preservation [21,22]. Contemporary recovery programmes prioritise early ambulation, rapid restoration of function, reduction in hospital length of stay, and maintenance of independence [23]. Within this framework, successful analgesia is no longer defined solely by lower pain scores but by the ability to facilitate safe mobilisation and functional recovery while minimising opioid-related adverse effects [24,25].

Therefore, the purpose of this narrative review is not simply to summarise available regional anaesthesia techniques for TKA, but to examine them through a geriatric perspective. We review the anatomical basis of motor-sparing analgesia, critically evaluate current evidence on established and emerging techniques, and propose a clinically oriented framework for selecting regional anaesthesia strategies based on frailty status, sarcopenia risk, mobility goals, and postoperative recovery priorities. Particular emphasis is placed on outcomes that are especially relevant to older adults, including preservation of quadricep function, fall prevention, mobilisation, delirium risk, and maintenance of functional independence.

## 2. Methods

For this narrative review, we searched PubMed, Scopus, and Google Scholar for peer-reviewed publications published between May 2014 and May 2026. Search concepts included “total knee arthroplasty”, “total knee replacement”, “regional anaesthesia”, “peripheral nerve block”, “motor-sparing nerve block”, “knee joint innervation”, “adductor canal block”, “iPACK block”, “frailty”, “sarcopenia”, “older adult*”, “aged”, and “geriatric”, combined with the Boolean operators AND and OR. Backward citation tracking was used to identify seminal anatomical and technique-related publications issued before May 2014.

Publications were considered relevant if they addressed: (1) the anatomical basis of sensory or motor blockade in TKA; (2) the analgesic or motor effects of a regional technique; or (3) outcomes relevant to older adults, including opioid consumption, time to mobilisation, gait or balance, falls or near-falls, quadricep function, range of motion, delirium, discharge destination, or recovery of independence. Systematic reviews, meta-analyses, randomised controlled trials, prospective or retrospective cohort studies, and clinically relevant anatomical studies were considered. Publications without TKA-specific or transferable anatomical or geriatric relevance were not retained. Significant eligibility restrictions were extracted for representative primary studies discussed in detail and are summarised in Table 3.

Study selection was purposive rather than exhaustive. Because this review was not prospectively registered or managed as a systematic review, stage-specific retrieval and exclusion counts were not recorded and a formal risk-of-bias assessment was not undertaken. The final narrative synthesis comprised 108 topic-specific publications; the SANRA publication [26] was cited separately as a methodological source. Two authors (PP, TR) independently screened titles and abstracts and assessed potentially relevant full texts. Disagreements regarding inclusion or evidence classification were resolved through discussion, with a third author (MR) acting as adjudicator when required.

Evidence was classified as geriatric-specific when all participants were aged ≥65 years or the results for that age group were reported separately; older-enriched mixed-age when younger adults remained eligible but the cohort mean or median age was approximately ≥65 years; and mixed-age adult when younger adults were eligible and no age-stratified results were provided. Application of older-enriched or mixed-age evidence to older adults—and especially to people with frailty or sarcopenia—was classified as extrapolation.

The strength of technique-specific evidence was categorised descriptively as high, moderate, limited, or very limited. High indicated replicated randomised evidence and/or support from systematic reviews or meta-analyses; moderate indicated clinical evidence from multiple studies with important inconsistency, indirectness, or limited independent replication; limited indicated one or two small or early clinical studies; and very limited indicated preliminary evidence or evidence based predominantly on anatomical rationale without adequate independent clinical validation. These categories represent narrative-review evidence tiers and should not be interpreted as a formal GRADE certainty assessment. Recommendations in the clinical decision framework were additionally labelled according to whether they were supported by geriatric-specific evidence, extrapolated mixed-age evidence, indirect evidence, or anatomical and geriatric reasoning.

Because of heterogeneity in techniques, comparators, populations, and outcome definitions, findings were synthesised narratively rather than pooled. This review was prepared and reported with reference to the Scale for the Assessment of Narrative Review Articles (SANRA) [26]; the author self-assessment is provided in Supplementary Table S1.

Before finalisation of this manuscript, the EQUATOR Network reporting-guideline library and guideline-selection resources were consulted [27]. No study-design-specific EQUATOR reporting guideline for general narrative reviews was identified. PRISMA and its extensions were not applied because the present article is a purposive narrative review rather than a systematic or scoping review. SANRA [26] was therefore used as the most directly applicable quality-assessment framework. Transferable principles of transparent reporting—including specification of the information sources and search period, eligibility principles, author involvement in publication selection, evidence classification, and methodological limitations—were nevertheless incorporated into the Methods.

## 3. Why Motor Preservation Matters in Older Adults Undergoing TKA

In older adults undergoing total knee arthroplasty, the clinical objective of postoperative analgesia extends beyond pain relief [28]. Effective pain control remains essential, but it must be achieved without compromising early mobilisation, balance, cognition, participation in rehabilitation, or functional independence [23,29]. In this population, even transient motor impairment may have consequences that are disproportionate to those observed in younger patients [29]. For this reason, preservation of quadricep strength should be considered a central component of perioperative analgesic planning rather than a secondary technical advantage [30].

### 3.1. Frailty

Frailty is a multidimensional syndrome characterised by reduced physiological reserve and increased vulnerability to surgical stress [31]. In the context of TKA, frailty is associated with higher rates of postoperative complications, prolonged hospitalisation, readmission, non-home discharge, and mortality [32]. Frailty has also been identified as an independent predictor of both acute and chronic postoperative pain [10,33]. It also reduces the patient’s capacity to tolerate complications that might otherwise be transient or clinically minor [34].

From an analgesic perspective, frailty is particularly relevant because it narrows the margin between effective pain control and functional harm. A regional technique that produces quadricep weakness may be acceptable in a robust adult, but in a frail older patient, it may delay mobilisation, increase dependence on assistance, and reduce the likelihood of discharge home [12,35,36]. Frail patients often have multiple comorbidities and polypharmacy, increasing their susceptibility to opioid-related adverse effects such as sedation, respiratory depression, constipation, urinary retention, and delirium [37,38].

Therefore, in frail patients, the ideal regional analgesic strategy should combine opioid-sparing efficacy with minimal motor impairment [39]. The goal is not simply to reduce pain intensity but to maintain sufficient strength, alertness, and balance to enable safe early mobilisation.

### 3.2. Sarcopenia

Sarcopenia, defined by reduced skeletal muscle mass, strength, and physical performance, is highly prevalent among older adults undergoing lower-limb arthroplasty [40]. Unlike frailty, which reflects global physiological vulnerability, sarcopenia directly affects the musculoskeletal reserve required for postoperative ambulation and rehabilitation [41].

This distinction is important for regional anaesthesia. TKA patients rely heavily on quadricep strength during transfers, standing, gait initiation, stair climbing, and physiotherapy. In sarcopenic patients, baseline quadricep reserve may already be critically reduced [42]. Any additional block-related motor weakness may therefore transform a manageable postoperative state into clinically relevant immobility [25,35].

Consequently, sarcopenia should be viewed as a strong argument against quadriceps-impairing techniques. Even partial weakness of the vastus medialis may be meaningful in patients with low preoperative muscle strength. In this subgroup, motor-sparing approaches are not merely preferable; they are functionally necessary [43]. Preoperative screening tools such as gait speed, grip strength, chair-rise testing, or the SARC-F questionnaire may help identify patients in whom motor preservation should be prioritised [40].

### 3.3. Delirium

Postoperative delirium is one of the most clinically important complications in older surgical patients [44]. It is associated with prolonged hospitalisation, increased institutional discharge, functional decline, cognitive deterioration, and mortality. After TKA, delirium risk is influenced by age, comorbidity burden, baseline cognitive impairment, sleep disruption, pain, opioid exposure, benzodiazepine use, and immobility [45].

Regional anaesthesia may contribute to delirium prevention through opioid-sparing analgesia and improved pain control. However, analgesic strategies that preserve cognition but impair mobility may still indirectly increase delirium risk by promoting bed rest, sleep fragmentation, dependence, and prolonged hospitalisation [46]. Thus, in older adults, delirium prevention requires a balanced approach: adequate analgesia, avoidance of excessive systemic opioids and sedatives, and preservation of mobility [47].

Motor-sparing regional techniques fit this objective particularly well. By reducing opioid requirements while allowing for early ambulation, they address two modifiable delirium-related pathways: pharmacological neurotoxicity and immobility-related functional decline [46,48].

### 3.4. Falls

Falls after TKA are clinically significant events that may result in wound complications, periprosthetic fracture, loss of confidence, delayed rehabilitation, prolonged hospitalisation, or institutional discharge [49]. Older adults are especially vulnerable because of impaired balance, reduced muscle strength, visual impairment, orthostatic hypotension, polypharmacy, and pre-existing gait instability [50].

Quadricep weakness is a major modifiable risk factor for falls after TKA [51]. Techniques such as femoral nerve block provide effective analgesia but may impair knee extension strength and reduce dynamic stability during early ambulation [17]. Although fall events are relatively infrequent and many trials are underpowered to detect them, near-falls, delayed mobilisation, and reduced quadricep strength are clinically meaningful surrogate outcomes [49].

For geriatric patients, fall prevention should be considered a core endpoint of regional analgesia [52]. The safest analgesic strategy is not necessarily the one that produces the lowest resting pain score, but the one that enables the patient to stand, transfer, and walk with preserved motor control [53]. This principle strongly favours sensory-predominant techniques such as adductor canal block, local infiltration analgesia, genicular nerve block, and posterior compartment blocks that avoid clinically significant motor impairment in the tibial, common fibular, or femoral nerves [54].

### 3.5. Functional Recovery

Functional recovery after TKA depends on the interaction among analgesia, muscle strength, motivation, cognition, access to rehabilitation, and baseline physical reserve [55]. Pain can limit participation in physiotherapy, but motor weakness can be equally restrictive. In older adults, the consequences of delayed mobilisation are amplified by lower physiological reserve and faster deconditioning [29].

Traditional analgesic outcomes, such as pain scores at rest and cumulative opioid consumption over 24–48 h, do not fully capture what matters most to geriatric patients [11]. More relevant outcomes include time to first ambulation, walking distance, ability to perform transfers, stair climbing, knee range of motion, discharge destination, need for inpatient rehabilitation, and recovery of independence in activities of daily living [29].

This shift in perspective has important implications for research and clinical practice. Regional anaesthesia studies in TKA should increasingly include mobility-centred and geriatric-relevant endpoints rather than focusing exclusively on pain intensity [56]. A block that produces a small analgesic advantage at the cost of measurable quadricep weakness may not represent a net benefit in frail or sarcopenic patients [57].

### 3.6. Enhanced Recovery After Surgery

Enhanced Recovery After Surgery pathways for TKA emphasise multimodal, opioid-sparing analgesia, early oral intake, rapid mobilisation, prevention of complications, and a shorter hospital stay [29]. Motor-sparing regional anaesthesia is highly aligned with these goals because it facilitates early physiotherapy while reducing reliance on systemic opioids [58].

Within ERAS protocols, the optimal block is not evaluated in isolation but as part of a broader recovery pathway [55]. Local infiltration analgesia, adductor canal block, iPACK, popliteal plexus block, and genicular nerve block may each contribute to analgesia while preserving the motor function required for same-day or next-day mobilisation [54]. Conversely, techniques associated with quadricep weakness may undermine ERAS objectives, particularly in older adults with frailty, sarcopenia, or high fall risk [29].

In geriatric TKA, therefore, motor preservation should be considered a prerequisite for successful enhanced recovery [58]. The most appropriate regional anaesthesia strategy is the one that supports the entire recovery trajectory: adequate analgesia, opioid minimisation, preserved strength, early ambulation, reduced risk of delirium, and safe discharge [28] (Table 1).

## 4. Anatomy Relevant to Motor-Sparing Analgesia

Successful motor-sparing analgesia after total knee arthroplasty requires a detailed understanding of the innervation of the knee [59]. Unlike many surgical procedures that involve a single dominant sensory pathway, postoperative pain after TKA originates from multiple structures—including skin, periosteum, ligaments, menisci, synovium, and the joint capsule—which receive overlapping innervation from the femoral, obturator, tibial, sciatic, and common fibular nerves [60]. Consequently, no single peripheral nerve block can reliably anaesthetise the entire surgical field.

From a clinical perspective, the knee can be divided into anterior and posterior compartments, each supplied by distinct sensory pathways and therefore requiring different analgesic approaches [61].

### 4.1. Anterior Knee Innervation

The anterior compartment is predominantly innervated by branches of the femoral nerve, with additional contributions from the obturator and sciatic nerve systems [59]. Anatomical studies have demonstrated that the anterior capsule receives sensory input through a network of nerves supplying four functional quadrants.

The superomedial quadrant is innervated primarily by the nerve to vastus medialis (NVM), the nerve to vastus intermedius (NVI), and the superomedial genicular nerve (SMGN) [62]. The NVM is particularly important because it carries both sensory fibres to the medial retinaculum and articular branches to the knee capsule while simultaneously providing motor innervation to the vastus medialis muscle [15]. This dual role explains why blocks performed near the femoral triangle may provide additional analgesia at the expense of partial quadricep weakness [63].

The superolateral quadrant receives contributions from the nerve to vastus lateralis (NVL), the lateral branch of the NVI, the superolateral genicular nerve (SLGN), and branches of the common fibular nerve. The inferomedial quadrant is supplied mainly by the inferomedial genicular nerve (IMGN), whereas the inferolateral quadrant is innervated by the inferolateral genicular nerve (ILGN) and the recurrent fibular nerve (RFN) [62]. Because of the close relationship between the ILGN and the common fibular nerve, interventions targeting the inferolateral compartment may carry a risk of inadvertent motor blockade and foot drop [64].

### 4.2. Posterior Knee Innervation

The posterior capsule is responsible for a substantial proportion of postoperative pain after TKA and is frequently inadequately covered by isolated anterior compartment blocks [61].

Its innervation originates primarily from articular branches of the tibial nerve, with additional contributions from the posterior branch of the obturator nerve, the sciatic nerve, and the common fibular nerve [65]. These branches form a dense sensory network known as the popliteal plexus [20]. Importantly, the popliteal plexus contains predominantly sensory fibres supplying the posterior capsule while remaining anatomically distinct from the major motor branches of the tibial and common fibular nerves [66].

**Figure 1:**
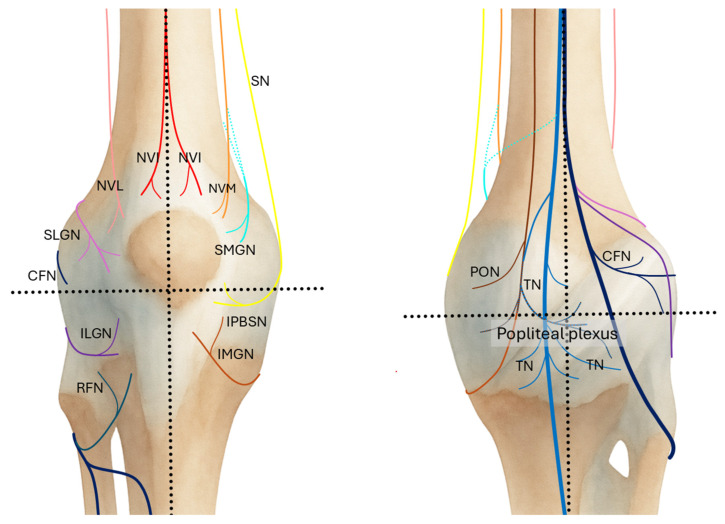
Schematic representation of knee innervation. Abbreviations: NVM = nerve to the vastus medialis; NVI = nerve to the vastus intermedius; NVL = nerve to the vastus lateralis; IPBSN = infrapatellar branch of the saphenous nerve; SMGN/SLGN/IMGN/ILGN = superomedial/superolateral/inferomedial/inferolateral genicular nerves; TN = tibial nerve; CFN = common fibular nerve; RFN = recurrent fibular nerve; SN = saphenous nerve; PON = posterior branch of obturator nerve.

This anatomical arrangement underpins posterior motor-sparing techniques such as iPACK and popliteal plexus block (PPB), both of which aim to anaesthetise posterior capsular afferents while preserving distal motor function [67,68] (Figure 1).

### 4.3. Cutaneous Innervation and the Surgical Incision

Although postoperative pain is often attributed primarily to deep articular structures, the midline surgical incision represents an important and frequently overlooked source of pain after TKA [69].

The distal portion of the incision is supplied predominantly by the infrapatellar branch of the saphenous nerve (IPBSN), which is routinely covered by an adductor canal block [15]. However, the proximal and central portions of the incision are innervated by the anterior femoral cutaneous nerve (AFCN), consisting of the medial femoral cutaneous nerve (MFCN) and intermediate femoral cutaneous nerve (IFCN) [16].

**Figure 2:**
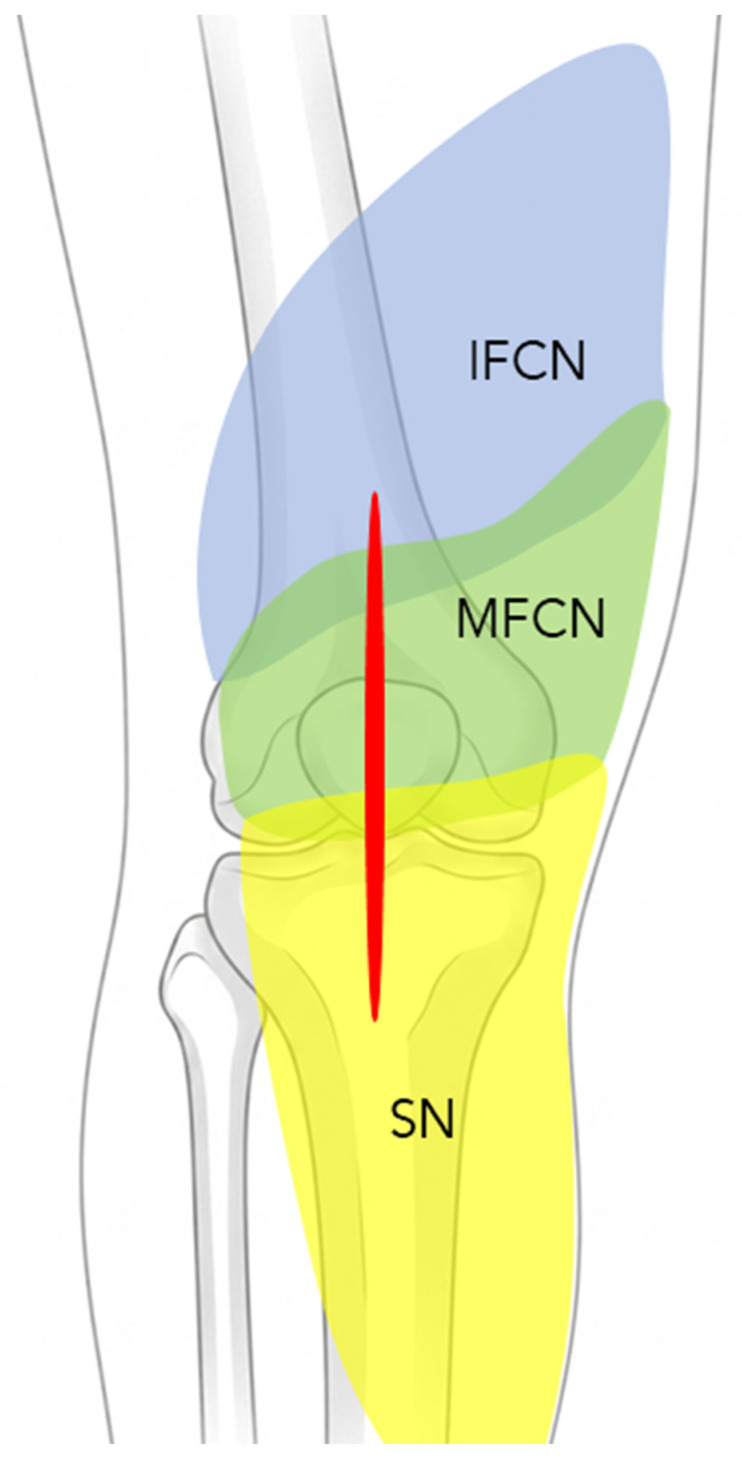
Skin innervation. Abbreviations: MFCN = medial femoral cutaneous nerve; IFCN = intermediate femoral cutaneous nerve; SN = saphenous nerve; red line = incision line.

This anatomical distinction explains why some patients continue to experience significant incisional pain despite a technically successful adductor canal block [63]. It also provides the anatomical basis for newer supplementary techniques such as anterior femoral cutaneous nerve block (AFCNB), which specifically targets sensory territories not routinely covered by conventional motor-sparing approaches [69] (Figure 2).

### 4.4. Anatomical Targets for Motor-Sparing Analgesia

From the perspective of postoperative function, not all neural targets are equally desirable. The central challenge of motor-sparing analgesia is to maximise sensory blockade while preserving quadricep function [14].

The most important sensory targets include the infrapatellar branch of the saphenous nerve, articular branches of the NVM, the medial and intermediate femoral cutaneous nerves, the genicular nerves, and the popliteal plexus [30]. In contrast, techniques performed too proximally may involve motor branches supplying the quadricep muscle, particularly the NVM and the femoral nerve itself [63].

This anatomical principle underlies the evolution of modern TKA analgesia. Contemporary motor-sparing strategies increasingly focus on selective blockade of distal sensory branches rather than proximal mixed nerves, thereby preserving quadricep strength while maintaining clinically meaningful analgesia [70]. Understanding these anatomical relationships is essential for interpreting the advantages and limitations of currently available regional anaesthesia techniques.

Table 2 summarises the relationship between the principal anatomical targets, corresponding regional techniques, expected sensory coverage, and potential risk of motor impairment.

## 5. Evidence-Informed Review of Motor-Sparing Techniques

The rapid evolution of regional anaesthesia for total knee arthroplasty (TKA) has led to multiple motor-sparing approaches with varying degrees of clinical validation [30]. Their value differs according to analgesic efficacy, functional implications, and the population studied. Unless a study is explicitly labelled as geriatric-specific below, the evidence derives from older-enriched mixed-age or mixed-age adult populations, and its application to frail or sarcopenic older adults is an extrapolation. Available techniques are categorised as established, emerging, experimental, or generally unsuitable for geriatric practice [79].

In this review, “established” denotes techniques supported by replicated randomised evidence and/or systematic reviews; “emerging” denotes techniques with promising clinical evidence but limited replication or population directness; and “experimental” denotes techniques supported mainly by small preliminary studies or anatomical rationale without adequate independent validation. These labels are pragmatic narrative descriptors rather than formal grades of recommendation. Classification as generally unsuitable for frail or sarcopenic patients reflects an unfavourable motor-risk profile rather than a lack of analgesic efficacy.

### 5.1. Established Techniques

#### 5.1.1. Adductor Canal Block (ACB)

ACB is widely regarded as the cornerstone of contemporary motor-sparing analgesia for total knee arthroplasty. By targeting the saphenous nerve and distal sensory branches of the femoral nerve while largely preserving quadricep function, ACB provides an effective balance between analgesia and early mobilisation [80].

Numerous randomised controlled trials and meta-analyses have demonstrated that ACB provides analgesia comparable to that of femoral nerve block (FNB) while significantly reducing quadricep weakness and facilitating earlier ambulation [63,81]. These advantages are particularly relevant in frail and sarcopenic older adults, in whom even transient motor impairment may delay rehabilitation, increase fall risk, and compromise functional recovery [82]. A representative fall-risk RCT enrolled 62 adults aged 18–80 years undergoing primary unilateral TKA (ASA I–III). Revision TKA, BMI ≥ 40 kg/m^2^, chronic opioid use, renal impairment or coagulopathy, and inability to provide informed consent were key exclusions [81]. ACB preserved quadricep strength better than FNB, but the study did not demonstrate a statistically significant reduction in high fall risk and was underpowered for rare fall events. No age-stratified, frailty, or sarcopenia analysis was reported; geriatric conclusions from this trial are therefore extrapolated.

Although ACB alone may not provide complete coverage of all nociceptive pathways involved in TKA, it serves as an effective foundation for multimodal motor-sparing analgesia [83]. Contemporary evidence supports its use in combination with local infiltration analgesia (LIA) and, when additional posterior coverage is required, with iPACK or popliteal plexus block (PPB). This combined approach aligns closely with Enhanced Recovery After Surgery (ERAS) principles by providing effective analgesia while preserving mobility [68,84].

From a geriatric perspective, the principal value of ACB lies not only in pain reduction but also in its ability to maintain quadricep strength and support early functional recovery [85]. Consequently, ACB remains the reference motor-sparing technique against which newer approaches are increasingly compared [15].

#### 5.1.2. Local Infiltration Analgesia (LIA)

Local infiltration analgesia has become a cornerstone of multimodal analgesia after TKA [17,86]. The technique involves periarticular infiltration of diluted local anaesthetic solutions during surgery and provides broad coverage of multiple nociceptive pathways without producing clinically significant motor weakness [74].

Evidence consistently demonstrates that LIA provides effective analgesia extending into the early postoperative period and supports rapid mobilisation. Its simplicity and surgeon-controlled administration make it widely applicable across diverse clinical settings [17].

The principal concern regarding LIA is the high doses of local anaesthetic commonly used. Although systemic toxicity remains uncommon, older adults may possess a narrower safety margin because of altered pharmacokinetics, reduced protein binding, and increased comorbidity burden [87]. Careful dose calculation and vigilance for local anaesthetic systemic toxicity (LAST) are therefore particularly important in geriatric populations [88].

Several systematic reviews suggest that combining LIA with ACB produces superior analgesia compared with either technique alone [89,90,91].

#### 5.1.3. Posterior Compartment Blocks: iPACK and Popliteal Plexus Block (PPB)

While ACB effectively addresses anterior knee pain, it provides limited coverage of the posterior capsule. Because posterior knee pain affects a substantial proportion of patients after TKA, supplementary posterior compartment blocks have gained increasing popularity [92].

The infiltration between the popliteal artery and the capsule of the knee (iPACK) block targets sensory branches of the popliteal plexus while preserving tibial and common fibular motor function [93]. Multiple systematic reviews and network meta-analyses have demonstrated improved analgesia and reduced opioid consumption when iPACK is combined with ACB, particularly when LIA is not administered [19,94]. One large RCT of iPACK + ACB versus sham enrolled 366 adults aged > 18 years undergoing elective primary unilateral TKA under spinal anaesthesia (ASA I–III) [68]. Opioid abuse, severe renal or hepatic failure, pre-existing lower-limb neurological disorders, and psychiatric or cognitive problems precluding reliable pain assessment were key exclusions. Range of motion and quadricep strength improved with iPACK + ACB, but age-specific, frailty, sarcopenia, falls, delirium, and independence outcomes were not reported; application to geriatric vulnerability is therefore extrapolated.

The popliteal plexus block (PPB) has emerged as a more anatomically selective alternative [20]. Current evidence suggests analgesic efficacy comparable to iPACK while preserving motor function [67]. An additional practical advantage is that PPB can often be performed without repositioning the patient, simplifying workflow. Despite growing interest in PPB, iPACK currently possesses a larger evidence base and remains the more established technique.

### 5.2. Emerging Techniques

#### 5.2.1. Genicular Nerve Block (GNB)

Genicular nerve block (GNB) has attracted considerable attention as a highly selective sensory technique targeting the terminal articular branches supplying the knee [18]. Unlike proximal nerve blocks, GNB directly addresses the nociceptive pathways responsible for postoperative pain while minimising the risk of motor impairment [73].

Recent meta-analyses demonstrate significant reductions in both resting and dynamic pain following TKA. An additional advantage is the markedly lower volume of local anaesthetic required compared with LIA, which may reduce the risk of systemic toxicity [64,73,95].

The long-term role of GNB remains uncertain. Questions persist regarding optimal targets, injection volumes, and integration into multimodal protocols. Nevertheless, current evidence suggests substantial clinical potential [96].

#### 5.2.2. Anterior Femoral Cutaneous Nerve Block (AFCNB)

Anatomical studies have demonstrated that conventional ACB does not reliably anaesthetise the entire TKA incision [16]. While the distal portion is supplied by the infrapatellar branch of the saphenous nerve, the central and proximal segments receive innervation from the anterior femoral cutaneous nerve [69]. AFCNB was developed specifically to address this gap. The available RCT analysed 79 adults aged ≤ 80 years (group means 61.9 and 65.2 years), with ASA I–III and BMI 18–40 kg/m^2^, undergoing elective primary TKA for osteoarthritis; severe deformity, chronic opioid use, and inability to cooperate were key exclusions [16]. Adding AFCNB to FTB reduced movement and incision pain, improved POD2 Timed Up and Go performance, and improved one POD0 quadricep measure. However, the trial was not geriatric-specific and did not stratify participants by frailty or sarcopenia; its geriatric applicability remains extrapolated.

### 5.3. Experimental Techniques

#### 5.3.1. Dual Subsartorial Block (DSB)

The dual subsartorial block combines injections within the distal femoral triangle and the adductor canal to broaden sensory coverage [71,97]. Early randomised data suggest improved analgesia and reduced opioid requirements compared with conventional approaches [98]. Despite encouraging results, evidence is currently limited to a small number of studies [99]. Furthermore, because one injection is performed near the vastus medialis nerve, theoretical concerns remain about subtle motor impairment [71].

#### 5.3.2. Triple-Injection Peri-Sartorius Block (TIPS)

TIPS extends the DSB concept by adding a third injection targeting the intermediate femoral cutaneous nerve. Preliminary studies have demonstrated improved dynamic pain control, lower opioid consumption, and enhanced functional recovery compared with more conventional approaches [72,98].

However, the evidence base remains extremely limited, and independent validation is lacking. As with DSB, the proximity of some injection sites to the vastus medialis nerve raises theoretical concerns regarding motor preservation [72].

#### 5.3.3. Erector Spinae Plane Block (ESPB)

The erector spinae plane block has recently been proposed as a motor-sparing alternative for TKA through indirect blockade of lumbar and sacral nerve roots. Theoretical advantages include technical simplicity and broad analgesic coverage [75,76,77].

Only two small, single-centre RCTs specifically evaluated ESPB in older adults undergoing TKA [75,76]. Reysner et al. reported 90 participants aged 65–89 years (ASA I–III) and excluded chronic pain, inability to assess pain, anticoagulation or bleeding diathesis, general anaesthesia, and non-standard implant or ligament-release procedures [76]. Pietraszek et al. enrolled 60 patients aged 65–100 years and excluded cognitive or communication impairment, chronic opioid use, operative-limb neuropathy, coagulopathy, infection, and local-anaesthetic contraindications [75]. Both trials found higher opioid requirements with ESPB than with iPACK + ACB while quadricep strength was preserved. Neither assessed frailty, sarcopenia, gait, falls, delirium, discharge destination, or activities of daily living, and independent replication is lacking. ESPB may be a rescue option when peripheral blocks are impractical, but current data do not support its routine first-line use.

### 5.4. Technique Requiring Caution When Motor Preservation Is Critical

#### Femoral Nerve Block (FNB)

For many years, femoral nerve block represented the standard regional analgesic technique for TKA [28]. Although it provides excellent analgesia, its principal limitation is predictable quadricep weakness resulting from blockade of motor fibres innervating the quadriceps [78].

In younger patients, this weakness is usually transient and clinically manageable. In frail or sarcopenic older adults, however, the consequences may be substantially greater. Reduced quadricep strength may delay mobilisation, increase dependence on assistance, impair participation in physiotherapy, and elevate the risk of falls [100].

While isolated studies suggest potential reductions in postoperative delirium with improved analgesia, these benefits do not outweigh the consistent evidence that FNB is associated with impaired functional recovery compared with motor-sparing alternatives [101] (Table 3 and Table 4).

## 6. Clinical Decision Framework for Frail and Sarcopenic Patients Undergoing TKA

Although numerous regional anaesthesia techniques have been described for total knee arthroplasty, evidence-informed guidance for selecting an optimal strategy in older adults remains limited [28]. Most available studies focus on analgesic efficacy rather than patient-specific factors such as frailty, sarcopenia, fall risk, cognitive vulnerability, or susceptibility to local anaesthetic toxicity [25,88,101,102,103]. Given the lack of dedicated randomised trials in these populations, the following framework synthesises current anatomical knowledge, available clinical evidence, and geriatric principles. Rather than prioritising pain scores alone, the proposed approach emphasises preservation of mobility, functional independence, and safe recovery (Table 5).

## 7. Key Clinical Messages

**Figure 3:**
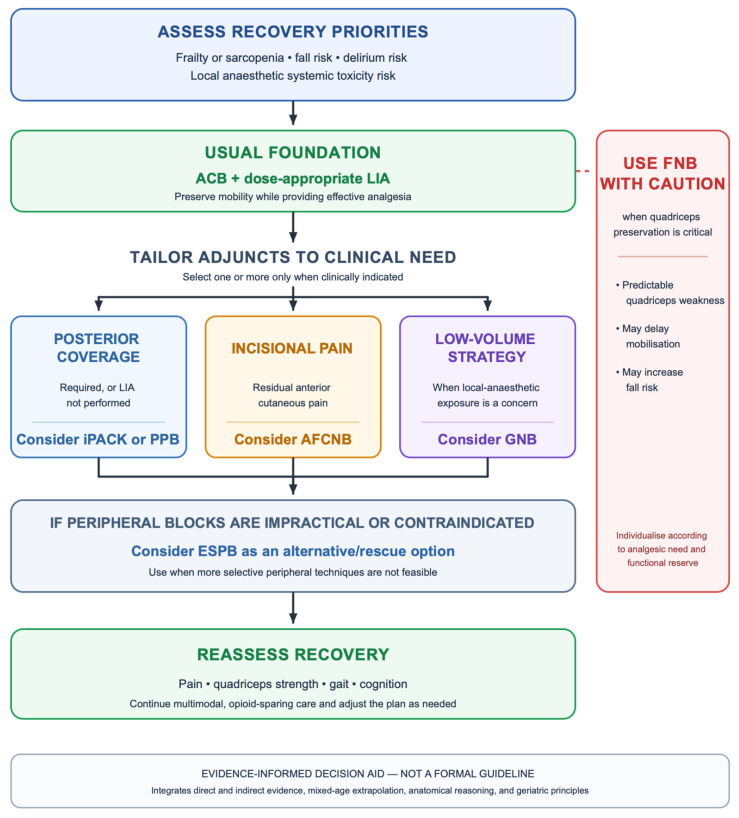
Simplified pragmatic decision aid for motor-sparing analgesia in older adults undergoing TKA. ACB with dose-appropriate LIA is the usual foundation, while posterior or cutaneous adjuncts should be selected based on the expected pain territory and individual risk profile. GNB may be considered when a low-volume strategy is desirable, and ESPB may serve as an alternative when peripheral blocks are impractical. FNB should be used cautiously when preservation of quadricep function is critical. This algorithm is evidence-informed and does not constitute a formal guideline.

First, ACB remains the cornerstone of motor-sparing analgesia and forms the basis of most recommended strategies [70,104]. Second, the preservation of quadricep strength should be prioritised in frail and sarcopenic patients, in whom even transient motor weakness may delay recovery and increase the risk of falls [53,62,105]. Third, GNB and AFCNB appear particularly attractive as supplementary techniques because they expand sensory coverage while maintaining a highly favourable motor profile [18,69]. Finally, although ESPB may provide useful analgesia when peripheral nerve blocks are impractical, current evidence does not support its routine use as a first-line motor-sparing strategy [75,76]. The proposed framework is summarised in Figure 3. It should be interpreted as a pragmatic decision aid rather than a formal guideline or prospectively validated algorithm, because its recommendations integrate direct and indirect clinical evidence, extrapolation from mixed-age cohorts, anatomical reasoning, and geriatric principles.

## 8. Knowledge Gaps and Future Directions

Despite the rapid development of motor-sparing regional anaesthesia techniques for total knee arthroplasty, significant gaps remain in the current evidence base [62]. Most available randomised controlled trials have focused on short-term analgesic outcomes, including pain scores, opioid consumption, and duration of analgesia [70]. While these endpoints are important, they may not adequately reflect recovery priorities in older adults, particularly those with frailty or sarcopenia [11,29].

A notable limitation is the paucity—not complete absence—of geriatric-specific trials. Two small single-centre RCTs reported cohorts composed exclusively of adults aged ≥65 years, but both evaluated ESPB against iPACK + ACB and neither assessed frailty, sarcopenia, gait, falls, delirium, discharge destination, or independence [75,76]. Most other trials were mixed-age or older-enriched mixed-age and frequently excluded cognitive impairment, chronic opioid use, severe comorbidity, or inability to complete outcome testing. Recommendations for frail and sarcopenic patients are therefore largely extrapolated from younger and physiologically selected cohorts.

Evidence regarding motor-sparing regional anaesthesia and postoperative delirium remains indirect [106,107]. Although opioid-sparing strategies may plausibly reduce delirium risk, no included geriatric-specific regional-block trial measured delirium, and no causal reduction can be claimed [107,108]. Future studies should use standardised delirium assessments and treat cognitive recovery as a prespecified outcome (Table 6).

## 9. Conclusions

On the basis of evidence derived predominantly from mixed-age adult populations, ACB combined with LIA appears to provide the most defensible foundation for contemporary motor-sparing analgesia after TKA. iPACK and PPB may be considered when additional posterior coverage is required. GNB and AFCNB are promising sensory-selective adjuncts, but their effectiveness and functional safety in patients with frailty or sarcopenia require prospective validation. DSB, TIPS, and ESPB should currently be regarded as investigational or alternative techniques rather than established geriatric standards. FNB may be less suitable when preservation of quadricep function is a primary recovery objective.

Dedicated trials stratified by frailty and sarcopenia are needed before population-specific recommendations can be considered definitive. Future studies should prioritise mobility, falls and near-falls, delirium, discharge destination, functional independence, and quality of life, in addition to pain and opioid consumption. The proposed approaches identify promising directions for such clinical trials but should not be interpreted as formal practice guidelines.

## Figures and Tables

**Table 1:** Traditional vs. Geriatric-Relevant Outcomes After TKA.

Outcome	Commonly Reported in TKA Analgesia Studies	Clinical Relevance in Older Adults
Pain score (VAS/NRS)	Yes	Moderate
Opioid consumption	Yes	Moderate
Rescue analgesia	Yes	Low–Moderate
Duration of analgesia	Yes	Low
Quadricep strength	Sometimes	High
Falls	Rarely	Very high
Near-falls	Rarely	Very high
Gait speed	Rarely	Very high
Walking distance	Rarely	High
Rehabilitation participation	Rarely	High
Delirium	Rarely	Very high
Discharge destination	Rarely	Very high
Functional independence	Rarely	Very high
Quality of life	Rarely	High

**Table 2:** Anatomical Targets, Corresponding Techniques, Sensory Coverage, and Potential Motor Risk.

Anatomical Target	Corresponding Technique(s)	Principal Sensory Territory	Potential Motor Impairment
Saphenous nerve and infrapatellar branch in the distal adductor canal [15,63]	ACB	Anteromedial knee, medial lower leg, distal incision, and part of the anterior capsule	Low; proximal injection or high-volume spread may involve branches associated with the NVM
Articular branches of the NVM and distal femoral triangle [16,62,71,72]	FTB, DSB, TIPS	Superomedial capsule and medial retinacular region	Moderate or uncertain; proximal spread may involve the motor component of the NVM
MFCN and IFCN [16,69]	AFCNB; cutaneous component of TIPS	Central and proximal portions of the surgical incision	Minimal; these are predominantly cutaneous sensory targets
Popliteal plexus and posterior capsular branches [20,65,66,67,68]	iPACK, PPB	Posterior capsule	Low when the injection remains selective; spread to the tibial or common fibular nerve may impair distal motor function
Genicular nerves, particularly SMGN, SLGN, and IMGN [62,64,73]	GNB	Periosteal and capsular sensory territories surrounding the femoral and tibial condyles	Very low for selective targets; the ILGN requires caution because of its proximity to the common fibular nerve
Periarticular tissues and surgical field [17,74]	LIA	Broad capsular, periosteal, and incisional coverage	Minimal direct motor risk; safety is influenced mainly by the total local-anaesthetic dose
Lumbar paraspinal fascial plane with indirect nerve-root spread [75,76,77]	ESPB	Diffuse and variably distributed analgesia; knee-specific coverage is indirect	Low in the available small trials, but spread and clinical effects remain unpredictable
Femoral nerve [63,78]	FNB	Broad anterior knee coverage	High; predictable quadricep weakness may delay mobilisation

Abbreviations: ACB, adductor canal block; AFCNB, anterior femoral cutaneous nerve block; DSB, dual subsartorial block; ESPB, erector spinae plane block; FNB, femoral nerve block; FTB, femoral triangle block; GNB, genicular nerve block; IFCN, intermediate femoral cutaneous nerve; ILGN, inferolateral genicular nerve; IMGN, inferomedial genicular nerve; iPACK, infiltration between the popliteal artery and capsule of the knee; LIA, local infiltration analgesia; MFCN, medial femoral cutaneous nerve; NVM, nerve to vastus medialis; PPB, popliteal plexus block; SLGN, superolateral genicular nerve; SMGN, superomedial genicular nerve; TIPS, triple-injection peri-sartorius block. Motor-risk categories are qualitative and reflect anatomical proximity and the available clinical evidence rather than formally estimated event rates.

**Table 3:** Geriatric-Relevant Outcomes, Eligibility Criteria, and Population Source in Representative Included Studies.

Geriatric-Relevant Outcome	Representative Study and Significant Eligibility Criteria	Population Source	Finding and Remaining Gap
Quadricep strength; opioid exposure	Pietraszek et al. [75], RCT, n = 60; age 65–100 years; elective primary TKA under spinal anaesthesia. Excluded cognitive/communication impairment, chronic opioid use, operative-limb neuropathy, coagulopathy/infection, and local-anaesthetic contraindications.	Geriatric-specific; frailty/sarcopenia not measured	MRC strength remained preserved in both groups; iPACK + ACB reduced 48 h MME versus two-level ESPB. Gait, falls, delirium, discharge destination, and ADL were not assessed.
Quadricep strength; opioid exposure	Reysner et al. [76], RCT, n = 90; reported cohort age 65–89 years, ASA I–III, primary OA/standard CR TKA. Excluded chronic pain, inability to assess pain, anticoagulation/bleeding diathesis, general anaesthesia, and non-standard surgery.	Geriatric-specific; frailty/sarcopenia not measured	No between-group difference in quadricep strength; iPACK + ACB reduced opioid use versus L-ESPB or no block. No gait, falls, delirium, discharge, or ADL outcomes.
Range of motion; quadricep strength	Domagalska et al. [68], RCT, n = 366; adults > 18 years, ASA I–III, elective primary unilateral TKA. Key exclusions included opioid abuse, severe organ failure, lower-limb neurological disorders, and inability to assess pain reliably.	Mixed-age adult; no age-stratified results	iPACK + ACB improved ROM and quadricep strength versus sham. Application to frailty and sarcopenia is extrapolated.
TUG; quadricep strength; length of stay	Kampitak et al. [16], RCT, n = 79 analysed; age ≤80 years, mean ages 61.9/65.2 years; ASA I–III, BMI 18–40. Excluded severe deformity, chronic opioid use, and inability to cooperate.	Older-enriched mixed-age; no ≥ s65 subgroup	AFCNB + FTB improved POD2 TUG and one POD0 quadricep measure; length of stay was similar. No frailty/sarcopenia stratification.
Fall risk; gait; quadricep strength	Elkassabany et al. [81], RCT, n = 62; age 18–80 years, primary unilateral TKA, ASA I–III. Excluded revision TKA, BMI ≥ 40, chronic opioid use, renal impairment/coagulopathy, and inability to consent.	Mixed-age adult; no older subgroup	ACB preserved quadricep strength; TUG and walking distance were similar, and high fall-risk proportions did not differ significantly. The trial was underpowered for actual falls.
Frailty and acute/chronic pain	Jin et al. [102], prospective cohort, n = 164; age ≥ 65 years, primary TKA; frailty measured using a comprehensive geriatric assessment-based frailty index.	Geriatric- and frailty-specific; no block comparison	Frailty independently predicted acute and chronic postsurgical pain. The study does not establish which regional technique improves geriatric functional outcomes.
Sarcopenia and functional recovery	Shon et al. [42], cohort, n = 445; 42 patients met AWGS 2019 sarcopenia criteria.	Sarcopenia-specific; not a regional-analgesia study	PROMs were worse through 6 months, except for pain, but not at 12 months. No comparison of blocks or early geriatric outcomes.
Delirium; discharge destination; ADL independence; long-term mobility	No included geriatric-specific regional-analgesia RCT reported these outcomes or stratified them by frailty or sarcopenia.	No direct evidence	Technique-specific effects on these priority outcomes remain unknown.

Geriatric-specific evidence was defined as an all- ≥65 cohort or a separately reported ≥65 subgroup. Older-enriched mixed-age evidence included younger adults but had a cohort mean or median age of approximately ≥65 years. Mixed-age adult evidence allowed for younger participants’ results and did not report separate older-adult results. Application of the latter two evidence categories to frailty or sarcopenia represents extrapolation.

**Table 4:** Evidence-Informed Hierarchy of Motor-Sparing Techniques for TKA.

Technique	Analgesic Efficacy	Motor Preservation	Evidence Tier	Role in Frail/Sarcopenic Patients
ACB	High	High	High	First-line
LIA	High	High	High	First-line
iPACK	Moderate-High	High	High	Adjunct
PPB	Moderate-High	High	Moderate	Adjunct
GNB	Moderate	Very high	Moderate	Promising
AFCNB	Moderate	Very high	Limited	Promising
DSB	Moderate	Uncertain	Very limited	Experimental
TIPS	Moderate	Uncertain	Very limited	Experimental
ESPB	Low-Moderate	High	Limited	Rescue option
FNB	High	Low	High	Use with caution; often less suitable when motor preservation is critical

ACB: adductor canal block; AFCNB: anterior femoral cutaneous nerve block; DSB: dual subsartorial block; ESPB: erector spinae plane block; FNB: femoral nerve block; GNB: genicular nerve block; iPACK: infiltration between the popliteal artery and capsule of the knee; LIA: local infiltration analgesia; PPB: popliteal plexus block; TIPS: triple-injection peri-sartorius block. Evidence tiers were assigned according to the descriptive criteria reported in the Methods and do not represent a formal GRADE assessment. The hierarchy integrates clinical evidence, population directness, anatomical reasoning, and geriatric recovery priorities.

**Table 5:** Proposed Pragmatic Clinical Decision Framework for Motor-Sparing Analgesia in Older Adults Undergoing TKA.

Clinical Scenario	Primary Goal	Preferred Strategy	Consider	Avoid/Use with Caution	Evidence Basis	Rationale
Standard older patient	Balance analgesia and mobilisation	ACB + LIA	ACB + iPACK/PPB	—	Mixed-age adult RCT/MA; extrapolated to routine ≥ 65 care	Most consistent evidence base for motor-sparing multimodal analgesia
Frailty	Preserve functional reserve and independence	ACB + LIA	PPB, GNB	FNB	Extrapolation; no frailty-specific regional-block RCT	Minimise motor impairment and opioid exposure; no dedicated frailty RCTs available
Sarcopenia	Preserve quadricep strength	ACB ± AFCNB	GNB	FNB, FTB, DSB, TIPS	Extrapolation; no sarcopenia-specific regional-block RCT	Even mild motor weakness may impair ambulation; no sarcopenia-specific RCTs
High fall risk	Maximise gait safety	ACB + LIA	AFCNB	FNB	Mixed-age adult RCT/MA; extrapolated; actual falls underpowered	Quadricep preservation is paramount; FNB is associated with falls in meta-analysis
High delirium risk	Reduce opioid consumption and promote early mobilisation	ACB + LIA	GNB, iPACK	Opioid-intensive strategies	Indirect adult evidence; no geriatric-specific delirium outcome	Delirium is linked to opioids and immobility; motor-sparing blocks address both
Persistent posterior knee pain	Improve posterior compartment analgesia	ACB + iPACK	PPB	Sciatic nerve block	Mixed-age RCT/MA plus two small ≥ 65 combination RCTs	Sensory posterior coverage with preserved tibial and fibular motor function
Persistent incision pain	Improve cutaneous analgesia	AFCNB	—	—	Older-enriched mixed-age RCT; no ≥65 subgroup	Covers MFCN/IFCN territories not reliably blocked by ACB
High LAST risk	Minimise local anaesthetic exposure	GNB	ACB	High-volume LIA	Mixed-age adult evidence; extrapolated	Small LA volumes may improve safety margin in older adults with altered pharmacokinetics
Peripheral block technically difficult or contraindicated	Provide alternative analgesia	ESPB	—	—	Two small geriatric-specific RCTs	Rescue option; two RCTs in the older adults show inferiority to ACB + iPACK—not first-line

ACB: adductor canal block; AFCNB: anterior femoral cutaneous nerve block; DSB: dual subsartorial block; ESPB: erector spinae plane block; FNB: femoral nerve block; FTB: femoral triangle block; GNB: genicular nerve block; iPACK: infiltration between the popliteal artery and capsule of the knee; LA: local anaesthetic; LAST: local anaesthetic systemic toxicity; LIA: local infiltration analgesia; MFCN: medial femoral cutaneous nerve; IFCN: intermediate femoral cutaneous nerve; PPB: popliteal plexus block; RCT: randomised controlled trial; MA: meta-analysis; TIPS: triple-injection peri-sartorius block. Evidence basis: High RCT/MA = supported by randomised controlled trials or meta-analyses; Extrapolation = inferred from mixed-age adult data; Low = limited to one or two small trials; Expert opinion = based on anatomical rationale and clinical reasoning.

**Table 6:** Knowledge Gaps in Motor-Sparing Analgesia for Frail and Sarcopenic Patients Undergoing TKA.

Domain	Current Evidence	Clinical Importance
Geriatric-specific regional-block RCTs	Two small ESPB-comparator RCTs; no frailty/sarcopenia stratification	Very high
Frailty-specific RCTs	None	Very high
Sarcopenia-specific RCTs	None	Very high
Delirium outcomes	Absent from geriatric-specific regional-block RCTs	High
Fall incidence	No adequately powered geriatric-specific RCT	High
Near-fall events	Rarely reported	High
Gait speed	Rarely reported	Very high
Functional independence	Absent from geriatric-specific block trials	Very high
Discharge destination	Absent from geriatric-specific block trials	Very high
Long-term mobility	Limited	Very high
Nursing home discharge	Absent	Very high
Cost-effectiveness	Absent	Moderate

Collectively, these gaps highlight a fundamental mismatch between the outcomes currently reported in TKA analgesia trials and those most relevant to geriatric patients [109]. Future studies should move beyond pain-centred endpoints and incorporate assessments of frailty, mobility measures, delirium outcomes, discharge destination, and long-term functional recovery [25,110]. Such an approach would provide clinicians with evidence that better reflects the priorities of older adults undergoing TKA.

## Data Availability

No new data were created or analysed in this study. Data sharing is not applicable to this article.

## Supplementary Materials

The following supporting information can be downloaded at: https://www.mdpi.com/article/10.3390/jcm15156071/s1, Supplementary Table S1. SANRA-based author self-assessment.

## Abbreviations

ACB	adductor canal block
AFCN	anterior femoral cutaneous nerve
AFCNB	anterior femoral cutaneous nerve block
C-ACB	continuous adductor canal block
DSB	dual subsartorial block
ERAS	enhanced recovery after surgery
ESPB	erector spinae plane block
FNB	femoral nerve block
FTB	femoral triangle block
GNB	genicular nerve block
IFCN	intermediate femoral cutaneous nerve
ILGN	inferolateral genicular nerve
IMGN	inferomedial genicular nerve
iPACK	infiltration between the popliteal artery and capsule of the knee
IPBSN	infrapatellar branch of the saphenous nerve
LA	local anaesthetic
LAST	local anaesthetic systemic toxicity
LIA	local infiltration analgesia
MA	meta-analysis
MFCN	medial femoral cutaneous nerve
NRS	numerical rating scale
NVI	nerve to the vastus intermedius
NVL	nerve to the vastus lateralis
NVM	nerve to the vastus medialis
PPB	popliteal plexus block
RCT	randomised controlled trial
RFN	recurrent fibular nerve
SARC-F	Strength, Assistance walking, Rise, Climb, Falls questionnaire
SLGN	superolateral genicular nerve
SMGN	superomedial genicular nerve
SN	saphenous nerve
TIPS	triple-injection peri-sartorius block
TKA	total knee arthroplasty
VAS	visual analogue scale

## References

[1] Geng R., Li J., Yu C., Zhang C., Chen F., Chen J., Ni H., Wang J., Kang K., Wei Z. (2023). Knee osteoarthritis: Current status and research progress in treatment. Exp. Ther. Med..

[2] Stubnya B.G., Schulz M., Váncsa S., Szilágyi G.S., Szatmári A., Bejek Z. (2025). Global Trends in Joint Arthroplasty: A Systematic Review and Future Projections. J. Clin. Med..

[3] Keast M., Hutchinson A.F., Khaw D., McDonall J. (2022). Impact of pain on postoperative recovery and participation in care following knee arthroplasty surgery: A qualitative descriptive study. Pain Manag. Nurs..

[4] Lavand’homme P.M., Kehlet H., Rawal N., Joshi G.P. (2022). Pain management after total knee arthroplasty: PROcedure SPEcific Postoperative Pain ManagemenT recommendations. Eur. J. Anaesthesiol..

[5] Chow R., Rajput K. (2024). Perioperative Pain Management: An Update on Pharmacology with a Special Focus on the Opioid Tolerant Elderly. Curr. Anesthesiol. Rep..

[6] Almodibeg B., Forget P. (2024). Challenges of acute pain management in older patients. Age Ageing.

[7] Ngcobo N.N. (2025). Influence of Ageing on the Pharmacodynamics and Pharmacokinetics of Chronically Administered Medicines in Geriatric Patients: A Review. Clin. Pharmacokinet..

[8] Shahbar A., Katlan D.A., Almutairy R.F., Alnoamy Y. (2024). Optimizing Medication Therapy in Elderly Patients: Therapeutic Management Challenges and Strategies. Difficulties and Challenges in Geriatric Health Management.

[9] D’Ambrosi R., Ursino C., Mariani I., Ursino N., Formica M., Chen A.F. (2023). Clinical outcomes, complications, and survivorship for unicompartmental knee arthroplasty versus total knee arthroplasty in patients aged 80 years and older with isolated medial knee osteoarthritis: A matched cohort analysis. Arch. Orthop. Trauma Surg..

[10] Jin Z., Rismany J., Gidicsin C., Bergese S.D. (2023). Frailty: The perioperative and anesthesia challenges of an emerging pandemic. J. Anesth..

[11] von Haehling S., Langer H.T., Heymsfield S.B., Evans W.J., Anker S.D. (2025). Sarcopenia in Ageing and Chronic Illness: Trial Endpoints and Regulatory Issues. J. Cachexia Sarcopenia Muscle.

[12] Wen H., Liu T., Li J. (2023). Association between frailty and clinical post-operative outcomes in patients following hip arthroplasty: A systematic review and meta-analysis. Int. Orthop..

[13] Kim Y.S., Choi S.I., Park J.Y., Yang H.W., Lee W., Kim S.H., Chung K., Kim N.Y. (2025). Low phase angle indicates poor muscle strength and physical performance in patients with knee osteoarthritis awaiting total knee arthroplasty. Sci. Rep..

[14] de Carvalho C.C., Pawa A., El-Boghdadly K. (2026). Motor-sparing regional anaesthesia in lower-limb arthroplasty: Balancing analgesia and function. Anaesthesia.

[15] Jiang X., Wang Q., Wu C., Tian W. (2016). Analgesic efficacy of adductor canal block in total knee arthroplasty: A meta-analysis and systematic review. Orthop. Surg..

[16] Kampitak W., Tanavalee A., Tansatit T., Ngarmukos S., Songborassamee N., Vichainarong C. (2021). The analgesic efficacy of anterior femoral cutaneous nerve block in combination with femoral triangle block in total knee arthroplasty: A randomized controlled trial. Korean J. Anesthesiol..

[17] Liu Q., Wang A., Zhang J. (2022). The effects of local infiltration anesthesia and femoral nerve block analgesia after total knee arthroplasty: A systematic review and meta-analysis. Ann. Transl. Med..

[18] Chang Y.S., Lee S.A., Wang H.L., Liao W.C. (2025). Genicular nerve block in people with total knee arthroplasty: A systematic review and meta-analysis. Medicine.

[19] Albrecht E., Wegrzyn J., Dabetic A., El-Boghdadly K. (2021). The analgesic efficacy of iPACK after knee surgery: A systematic review and meta-analysis with trial sequential analysis. J. Clin. Anesth..

[20] Sørensen J.K., Grevstad U., Jaeger P., Nikolajsen L., Runge C. (2025). Effects of popliteal plexus block after total knee arthroplasty: A randomized clinical trial. Reg. Anesth. Pain Med..

[21] Wainwright T.W., Gill M., McDonald D.A., Middleton R.G., Reed M., Sahota O., Yates P., Ljungqvist O. (2020). Consensus statement for perioperative care in total hip replacement and total knee replacement surgery: Enhanced Recovery After Surgery (ERAS^®^) Society recommendations. Acta Orthop..

[22] Albrecht E., Koninckx A., Kull C., Oliveira M., Addor V., Rossel J.B., Wegrzyn J. (2026). Effect of an enhanced recovery after surgery program on total hip and knee arthroplasty in a university hospital: A two-cohort study. Int. Orthop..

[23] Morrell A.T., Layon D.R., Scott M.J., Kates S.L., Golladay G.J., Patel N.K. (2021). Enhanced recovery after primary total hip and knee arthroplasty: A systematic review. JBJS.

[24] Courtine M., Bourredjem A., Gouteron A., Fournel I., Bartolone P., Baulot E., Ornetti P., Martz P. (2023). Functional recovery after total hip/knee replacement in obese people: A systematic review. Ann. Phys. Rehabil. Med..

[25] Hung M., Jensen A., Strickler I., Vu S., Hon E.S., Arapovic A., El-Othmani M.M. (2026). Frailty and Sarcopenia as Predictors of Functional Recovery After Total Hip and Knee Arthroplasty: A Narrative Review. J. Clin. Med..

[26] Baethge C., Goldbeck-Wood S., Mertens S. (2019). SANRA—A Scale for the Assessment of Narrative Review Articles. Res. Integr. Peer Rev..

[27] EQUATOR Network. Reporting Guidelines. https://www.equator-network.org/reporting-guidelines/.

[28] Xue X., Lv X., Ma X., Zhou Y., Yu N. (2024). Postoperative pain relief after total knee arthroplasty: A Bayesian network meta-analysis and systematic review of analgesic strategies based on nerve blocks. J. Clin. Anesth..

[29] He P., Xu Q., Feng Y. (2026). Assessment of enhanced recovery after surgery protocol in older adults undergoing total knee arthroplasty. Medicine.

[30] Layera S., Aliste J., Bravo D., Saadawi M., Salinas F.V., Tran D.Q. (2021). Motor-sparing nerve blocks for total knee replacement: A scoping review. J. Clin. Anesth..

[31] Clegg A., Young J., Iliffe S., Rikkert M.O., Rockwood K. (2013). Frailty in elderly people. Lancet.

[32] Partridge J.S., Ryan J., Dhesi J.K. (2022). New guidelines for the perioperative care of people living with frailty undergoing elective and emergency surgery—A commentary. Age Ageing.

[33] Tummala S., Mittal M.M., Sambandam S.N., Chen A.F., Wukich D.K. (2026). Impact of Frailty on Total Knee Arthroplasty Outcomes: A Propensity-Matched Study of 133,264 Patients Using the Modified Frailty Index. J. Arthroplast..

[34] Issa T.Z., Reynolds C.A., Dooley J.H., Thomas W.C., Sontag-Milobsky I., Hardt K.D., Manning D.W. (2025). Utility of Frailty index in predicting discharge disposition and prolonged length of stay following enhanced recovery after surgery protocol total hip and knee arthroplasty. Arthroplast. Today.

[35] Mathai A.S., Joy M., Varghese S.M., Koshy I. (2026). Frailty and sarcopenia as predictors of postoperative outcomes in elderly patients undergoing lower limb orthopedic surgeries in South India. J. Anaesthesiol. Clin. Pharmacol..

[36] Howell S.J., Nair S. (2021). Measuring frailty in the older surgical patient: The case for evidence synthesis. Br. J. Anaesth..

[37] Liu E.X., Kuhataparuks P., Liow M.H.L., Pang H.N., Tay D.K.J., Chia S.L., Lo N., Yeo S., Chen J.Y. (2023). Clinical Frailty Scale is a better predictor for adverse post-operative complications and functional outcomes than Modified Frailty Index and Charlson Comorbidity Index after total knee arthroplasty. Knee Surg. Sports Traumatol. Arthrosc..

[38] Penfold C.M., Judge A., Sayers A., Whitehouse M.R., Wilkinson J., Blom A.W. (2022). Temporal trends in comorbidity in adult elective hip and knee arthroplasty patients in England: A national population-based cohort study from the National Joint Registry. Bone Jt. J..

[39] Lee K.H., Chang W.L., Tsai S.W., Chen C.F., Wu P.K., Chen W.M. (2023). The impact of Charlson Comorbidity Index on surgical complications and reoperations following simultaneous bilateral total knee arthroplasty. Sci. Rep..

[40] Pegreffi F., Chiaramonte R., Donati Zeppa S., Lauretani F., Salvi M., Zucchini I., Veronese N., Vecchio M., Bartolacci A., Stocchi V. (2024). Optimizing the preoperative preparation of sarcopenic older people: The role of prehabilitation and nutritional supplementation before knee arthroplasty. Nutrients.

[41] Longo U.G., De Salvatore S., Borredon A., Manon K.Y., Marchetti A., De Marinis M.G., Denaro V. (2023). The effects of sarcopenia on hip and knee replacement surgery: A systematic review. Medicina.

[42] Shon O.J., Kim G.B., Cho S.J. (2023). Does sarcopenia accompanying end-stage knee osteoarthritis affect the outcomes following total knee arthroplasty?. Medicina.

[43] Chang K., Albright J.A., Testa E.J., Balboni A.B., Daniels A.H., Cohen E. (2023). Sarcopenia is associated with an increased risk of postoperative complications following total hip arthroplasty for osteoarthritis. Biology.

[44] Weinstein S.M., Poultsides L., Baaklini L.R., Mörwald E.E., Cozowicz C., Saleh J.N., Arrington M.B., Poeran J., Zubizarreta N., Memtsoudis S.G. (2018). Postoperative delirium in total knee and hip arthroplasty patients: A study of perioperative modifiable risk factors. Br. J. Anaesth..

[45] Kitsis P., Zisimou T., Gkiatas I., Kostas-Agnantis I., Gelalis I., Korompilias A., Pakos E. (2022). Postoperative delirium and postoperative cognitive dysfunction in patients with elective hip or knee arthroplasty: A narrative review of the literature. Life.

[46] Ni Y., Yang X., Yang Y., Zhang H., Peng S. (2025). Incidence and factors associated with postoperative delirium after primary total joint arthroplasty in older adults: A systematic review and meta-analysis. Front. Med..

[47] Callan K.T., Donnelly M., Lung B., McLellan M., DiGiovanni R., McMaster W., Yang S., Stitzlein R. (2024). Risk factors for postoperative delirium in orthopaedic hip surgery patients: A database review. BMC Musculoskelet. Disord..

[48] He Z., Kangjie K., Huang Z., Fang J. (2022). Effect of reducing-opioids consumption on postoperative delirium incidence in elderly patients after gastric cancer surgery. Zhonghua Yi Xue Za Zhi.

[49] Lawrence K.W., Link L., Lavin P., Schwarzkopf R., Rozell J.C. (2024). Characterizing patient factors, perioperative interventions, and outcomes associated with inpatients falls after total knee arthroplasty. Knee Surg. Relat. Res..

[50] Sadiq S., Noor R., Akram R. (2024). Risk factors of post-discharge falls in patients undergoing total knee arthroplasty: An integrative review. J. Back Musculoskelet. Rehabil..

[51] Kubo Y., Fujita D., Sugiyama S., Takachu R., Sugiura T., Sawada M., Yamashita K., Kobori K., Kobori M. (2024). Quadriceps strength loss following total knee arthroplasty as a predictor of three-month strength recovery: A secondary analysis of a randomized controlled trial. Cureus.

[52] Elliott M.R. (2023). Surrogate endpoints in clinical trials. Annu. Rev. Stat. Its Appl..

[53] Sun X.J., Feng T.C., Wang Y.M., Wang F., Zhao J.B., Liu X., Li F.L. (2023). The effect of the enhanced recovery after surgery protocol and the reduced use of opioids on postoperative outcomes in elderly patients with colorectal cancer. Eur. Rev. Med. Pharmacol. Sci..

[54] Fillingham Y.A., Hannon C.P., Kopp S.L., Austin M.S., Sershon R.A., Stronach B.M., Meneghini R.M., Abdel M.P., Griesemer M.E., Woznica A. (2022). The efficacy and safety of regional nerve blocks in total knee arthroplasty: Systematic review and direct meta-analysis. J. Arthroplast..

[55] Boekesteijn R., Smolders J., Busch V., Keijsers N., Geurts A., Smulders K. (2022). Objective monitoring of functional recovery after total knee and hip arthroplasty using sensor-derived gait measures. PeerJ.

[56] Choi J.Y., Rajaguru V., Shin J., Kim K.I. (2023). Comprehensive geriatric assessment and multidisciplinary team interventions for hospitalized older adults: A scoping review. Arch. Gerontol. Geriatr..

[57] Kong C., Zhang Y., Wang C., Wang P., Li X., Wang W., Wang Y., Shen J., Ren X., Wang T. (2022). Comprehensive geriatric assessment for older orthopedic patients and analysis of risk factors for postoperative complications. BMC Geriatr..

[58] Liimakka A.P., Farid A.R., Zhu L., Monette P.J., Varady N.H., Lange J.K., Javedan H., Chen A.F. (2025). Perioperative Geriatrician Assessment Is Associated with a Lower Risk of Emergency Department Visits After Total Joint Arthroplasty. JBJS.

[59] Suresh R., Buddhiraju A., Kuo K., Dellon A.L., Tuffaha S., Williams E. (2025). A cadaveric study of the innervation of the anterior compartment of the knee. Arch. Orthop. Trauma Surg..

[60] Obeidat A., Li J., Carter R.G., Olmer M., Fullam S., Mehta B., Otero M., Ramirez D., Miller R.J., Orange D. (2025). Mapping the Synovial Innervation of the Human Knee Joint. Osteoarthr. Cartil..

[61] Jorge P.B., de Oliveira D.E., de Resende V.R., Horita M.M., de Oliveira E Silva M., Duarte A., Santili C., Guglielmetti L.G.B. (2022). Knee anteromedial compartment dissection: Final results and anterior oblique ligament description. J. Orthop. Res..

[62] White L., Kerr M., Thang C., Pawa A. (2025). Motor-sparing regional anaesthesia for total knee arthroplasty: A narrative and systematic literature review. Br. J. Anaesth..

[63] Jæger P., Nielsen Z.J., Henningsen M.H., Hilsted K.L., Mathiesen O., Dahl J.B. (2013). Adductor canal block versus femoral nerve block and quadriceps strength: A randomized, double-blind, placebo-controlled, crossover study in healthy volunteers. Surv. Anesthesiol..

[64] Belba A., Peene L., Mesotten D., Vanlommel L., Van Zundert J., Vanneste T. (2026). The GENIFEM Pilot Randomized Trial: Genicular Nerve Block vs Femoral Triangle Block vs Local Infiltration Analgesia for Total Knee Arthroplasty. Anesth. Analg..

[65] Mifsut-Aleixandre M., Mifsut D., González-Soler E.M., Blasco-Serra A., Valverde A.A. (2024). Anatomical Safety Area for Periarticular Analgesic Infiltration through the Posterior Capsule in Total Knee Arthroplasty: Radiological Study in Magnetic Resonance. J. Clin. Med..

[66] Chan E., Howle R., Onwochei D., Desai N. (2021). Infiltration between the popliteal artery and the capsule of the knee (IPACK) block in knee surgery: A narrative review. Reg. Anesth. Pain Med..

[67] Stebler K., Elia N., Zaccaria I., Fournier R.M. (2026). Popliteal plexus block in total knee arthroplasty: A single-center randomized controlled double-blinded trial. Reg. Anesth. Pain Med..

[68] Domagalska M., Reysner T., Kowalski G., Daroszewski P., Mularski A., Wieczorowska-Tobis K. (2023). Pain Management, Functional Recovery, and Stress Response Expressed by NLR and PLR after the iPACK Block Combined with Adductor Canal Block for Total Knee Arthroplasty—A Prospective, Randomised, Double-Blinded Clinical Trial. J. Clin. Med..

[69] Bjørn S., Nielsen T.D., Moriggl B., Hoermann R., Bendtsen T.F. (2020). Anesthesia of the anterior femoral cutaneous nerves for total knee arthroplasty incision: Randomized volunteer trial. Reg. Anesth. Pain Med..

[70] Hussain N., Brull R., Vannabouathong C., Robinson C., Zhou S., D’Souza R.S., Sawyer T., Terkawi A.S., Abdallah F.W. (2023). Analgesic Effectiveness of Motor-Sparing Nerve Blocks for Total Knee Arthroplasty: A Network Meta-Analysis. Anesthesiology.

[71] Sonawane K., Dixit H., Mistry T., Balavenkatasubramanian J. (2021). Anatomical and technical considerations of “Dual Subsartorial Block” (DSB), a novel motor-sparing regional analgesia technique for total knee arthroplasty. Open J. Orthop. Rheumatol..

[72] Alabd A.S., Moustafa M.A., Ahmed A.M.M. (2024). Triple injection peri-sartorius (TIPS) block for postoperative analgesia after total knee arthroplasty: Randomised controlled study. Indian J. Anaesth..

[73] Eid G.M., El Said Shaban S., Mostafa T.A. (2023). Comparison of ultrasound-guided genicular nerve block and knee periarticular infiltration for postoperative pain and functional outcomes in knee arthroplasty—A randomised trial. Indian J. Anaesth..

[74] Schubert A.K., Seneviratne V., Stolz J., Wiesmann T., Wulf H., Eberhart L., Dinges H.C. (2023). The effect of adjuvants added to local anaesthetics for single-injection upper extremity peripheral regional anaesthesia: A systematic review with network meta-analysis of randomised trials. Eur. J. Anaesthesiol. EJA.

[75] Pietraszek P., Reysner T., Gola W., Kluzik A., Perek A., Marszałek-Buko J., Reysner M. (2026). Combined iPACK and adductor canal block versus two-level erector spinae plane block in elderly patients undergoing total knee arthroplasty: A randomized, triple-blinded clinical trial. Arch. Orthop. Trauma Surg..

[76] Reysner T., Wieczorowska-Tobis K., Kowalski G., Lapaj L., Daroszewski P., Reysner M. (2025). IPACK block with adductor canal block vs. lumbar erector spinae plane block (L-ESPB) in total knee arthroplasty: A randomized, double-blinded, controlled trial. Anaesthesiol. Intensive Ther..

[77] Ayub A., Talawar P., Gupta S.K., Kumar R., Alam A. (2019). Erector spinae plane block: A safe, simple and effective alternative for knee surgery. Anaesth. Intensive Care.

[78] Yang Y., Xu Y., Tang H., Hu Y., Bai F. (2025). The efficacy and safety of peripheral nerve blocks for postoperative analgesia following total hip arthroplasty: A network meta-analysis. BMC Anesthesiol..

[79] Kappenschneider T., Maderbacher G., Weber M., Greimel F., Holzapfel D., Parik L., Schwarz T., Leiss F., Knebl M., Reinhard J. (2022). Special orthopaedic geriatrics (SOG)-a new multiprofessional care model for elderly patients in elective orthopaedic surgery: A study protocol for a prospective randomized controlled trial of a multimodal intervention in frail patients with hip and knee replacement. BMC Musculoskelet. Disord..

[80] Manickam B., Perlas A., Duggan E., Brull R., Chan V.W., Ramlogan R. (2009). Feasibility and efficacy of ultrasound-guided block of the saphenous nerve in the adductor canal. Reg. Anesth. Pain Med..

[81] Elkassabany N.M., Antosh S., Ahmed M., Nelson C., Israelite C., Badiola I., Cai L.F., Williams R., Hughes C., Mariano E.R. (2016). The risk of falls after total knee arthroplasty with the use of a femoral nerve block versus an adductor canal block: A double-blinded randomized controlled study. Anesth. Analg..

[82] Deiter J., Ponzio D., Grau L., Griffiths S., Ong A., Post Z., Doucette D., Orozco F. (2020). Efficacy of adductor canal block protocol implementation in a multimodal pain management protocol for total knee arthroplasty. J. Clin. Orthop. Trauma.

[83] Tan Z., Kang P., Pei F., Shen B., Zhou Z., Yang J. (2018). A comparison of adductor canal block and femoral nerve block after total-knee arthroplasty regarding analgesic effect, effectiveness of early rehabilitation, and lateral knee pain relief in the early stage. Medicine.

[84] Mou P., Wang D., Tang X.M., Zeng W.N., Zeng Y., Yang J., Zhou Z.-K. (2022). Adductor canal block combined with IPACK block for postoperative analgesia and function recovery following total knee arthroplasty: A prospective, double-blind, randomized controlled study. J. Arthroplast..

[85] Hasabo E.A., Assar A., Mahmoud M.M., Abdalrahman H.A., Ibrahim E.A., Hasanin M.A., Emam A.K., AbdelQadir Y.H., AbdelAzim A.A., Ali A.S. (2022). Adductor canal block versus femoral nerve block for pain control after total knee arthroplasty: A systematic review and meta-analysis. Medicine.

[86] Juszkiewicz S., Juszkiewicz A., Kotrych D. (2019). The application of local infiltration analgesia as multimodal analgesia in postoperative pain managment after total knee arthroplasty—Review of the literature. Chir. Nsrzodow Ruchu Ortop. Pol..

[87] Tsai Y.F., Lin Y.C., Hsieh P.H., Liou J.T., Chung Y.T., Shih B.F., Yang M.-W., Liu F.-C., Lin H.-T. (2024). Incidence of local anesthetic systemic toxicity in patients receiving bupivacaine infiltration analgesia for total joint arthroplasty under general anesthesia: A retrospective single-center study. BMC Anesthesiol..

[88] Lungonyonyi R.K., Bleuze P., Boutière J.P. (2022). Local anesthetic systemic toxicity after local infiltration analgesia following a total knee arthroplasty. Cureus.

[89] Mott A., Brady S., Briggs I., Barrett M., Fulbright H., Hamilton T.W., Hewitt C., Palan J., Pandit H. (2024). Pain control post total knee replacement in patients given local infiltrative analgesia combined with adductor canal block compared to either modality alone: A systematic review and meta-analysis. BMJ Open.

[90] Xing Q., Dai W., Zhao D., Wu J., Huang C., Zhao Y. (2017). Adductor canal block with local infiltrative analgesia compared with local infiltrate analgesia for pain control after total knee arthroplasty: A meta-analysis of randomized controlled trials. Medicine.

[91] Lv J., Huang C., Wang Z., Ou S. (2020). Adductor canal block combined with local infiltration analgesia versus isolated adductor canal block in reducing pain and opioid consumption after total knee arthroplasty: A systematic review and meta-analysis. J. Int. Med. Res..

[92] Guo J., Hou M., Shi G., Bai N., Huo M. (2022). IPACK block (local anesthetic infiltration of the interspace between the popliteal artery and the posterior knee capsule) added to the adductor canal blocks versus the adductor canal blocks in the pain management after total knee arthroplasty: A systematic review and meta-analysis. J. Orthop. Surg..

[93] Biehl M., Wild L., Waldman K., Haq F., Easteal R.A., Sawhney M. (2020). The safety and efficacy of the IPACK block in primary total knee arthroplasty: A retrospective chart review. Can. J. Anesth. Can. Anesth..

[94] Bh P.P., Jinadu S., Okunlola O., Darkzali H., Lin H.M., Lai Y.H. (2023). Integrating IPACK (interspace between the popliteal artery and capsule of the posterior knee) block in an Enhanced Recovery after Surgery pathway for total knee arthroplasty—A prospective triple-blinded randomized controlled trial. J. Knee Surg..

[95] Cuñat T., Mejía J., Tatjer I., Comino O., Nuevo-Gayoso M., Martín N., Tió M., Basora M., Sala-Blanch X. (2023). Ultrasound-guided genicular nerves block vs. local infiltration analgesia for total knee arthroplasty: A randomised controlled non-inferiority trial. Anaesthesia.

[96] Küçükalp A., Özdemir B. (2023). Pain management following simultaneous bilateral total knee arthroplasty: Genicular nerve blockade versus periarticular injection. Acta Orthop. Belg..

[97] Li D., Alqwbani M., Wang Q., Yang Z., Liao R., Kang P. (2021). Ultrasound-guided adductor canal block combined with lateral femoral cutaneous nerve block for post-operative analgesia following total knee arthroplasty: A prospective, double-blind, randomized controlled study. Int. Orthop..

[98] Moustafa M.A., Alabd A.S., Ahmed A.M.M. (2026). Triple-injection perisartorius block versus dual subsartorial block for total knee arthroplasty: A double-blinded randomized controlled study. Anesth. Pain Med..

[99] Desai D., Shah N., Choube K. (2025). Comparative analysis of dual versus single subsartorial block for postoperative analgesia after total knee arthroplasty—A randomized, double-blind study. J. Anaesthesiol. Clin. Pharmacol..

[100] Reysner T., Kowalski G., Mularski A., Reysner M., Wieczorowska-Tobis K. (2025). From Pain Control to Early Mobility: The Evolution of Regional Anesthesia in Geriatric Total Hip Arthroplasty. Reports.

[101] Wu K.A., Kutzer K.M., Kugelman D.N., Seyler T.M. (2025). Fall prevention after hip and knee arthroplasty. Orthop. Clin..

[102] Jin Y., Tang S., Wang W., Zhang W., Hou Y., Jiao Y., Hou B., Ma Z. (2024). Preoperative frailty predicts postoperative pain after total knee arthroplasty in older patients: A prospective observational study. Eur. Geriatr. Med..

[103] Ren S., Yuan F., Yuan S., Zang C., Zhang Y., Lang B. (2022). Early cognitive dysfunction in elderly patients after total knee arthroplasty: An analysis of risk factors and cognitive functional levels. BioMed Res. Int..

[104] Fan Chiang Y.H., Wang M.T., Chan S.M., Chen S.Y., Wang M.L., Hou J.D., Tsai H.-C., Lin J.-A. (2023). Motor-Sparing Effect of Adductor Canal Block for Knee Analgesia: An Updated Review and a Subgroup Analysis of Randomized Controlled Trials Based on a Corrected Classification System. Healthcare.

[105] de Carvalho C.C., El-Boghdadly K., Guedes I.H.L., Dantas M.V.M., Tomé D.P.B., Ramos I.B., Gomes C.S.H., Alves G.K.P.A., Bezerra A.P., Santos Neto J.M. (2026). Opioid-free vs. opioid-inclusive anaesthesia with or without regional anaesthesia for postoperative pain: A systematic review with network meta-analysis of randomised controlled trials. Anaesthesia.

[106] Yasin M.B., Raheel M., Ahmed M.M., Ashfaq S., Asim H.S., Rauf M.Q., Fahim R., Chhetri R. (2026). Impact of preoperative frailty on complications, readmissions, and functional recovery following total knee arthroplasty in elderly patients. World J. Orthop..

[107] Zhao J., Liang G., Hong K., Pan J., Luo M., Liu J., Huang B. (2022). Risk factors for postoperative delirium following total hip or knee arthroplasty: A meta-analysis. Front Psychol..

[108] Zhang Y., Yu Y., Han Z., Diao L., Zhao R., Liu J., Luod Y., Wua H., Yang Y. (2024). Incidence and associated factors of delirium after primary total joint arthroplasty in elderly patients: A systematic review and meta-analysis. Medicine.

[109] Disalvo D., Moth E., Soo W.K., Garcia M.V., Blinman P., Steer C., Amgarth-Duff I., Power J., Phillips J., Agar M. (2023). The effect of comprehensive geriatric assessment on care received, treatment completion, toxicity, cancer-related and geriatric assessment outcomes, and quality of life for older adults receiving systemic anti-cancer treatment: A systematic review. J. Geriatr. Oncol..

[110] Peters X.D., Russell M.M. (2024). Patient Centered Outcomes After Surgery in the Older Adult. Curr. Geriatr. Rep..

