# Supplementary material for: Motor-Sparing Regional Analgesia in Frail and Sarcopenic Older Adults Undergoing Total Knee Arthroplasty: From Anatomy to Clinical Decision-Making—A Narrative Review
Source: J Clin Med. 2026 Aug 4;15(15):6071. doi: 10.3390/jcm15156071 (PMC13466388; doi:10.3390/jcm15156071)
Supplement: Supplementary file 1 [file jcm-15-06071-s001.zip › jcm-4452460-supplementary.pdf]

Supplementary Table S1. SANRA-based author self-assessment.

SANRA item	Score	Basis
Justification of the article’s importance	2	Importance for older adults, frailty, sarcopenia, and mobility is explained in the Introduction and Section 3.
Statement of concrete aims	2	The aims are stated in the Abstract and final Introduction paragraph.
Description of the literature search	2	Databases, dates, concepts, study types, outcomes, citation tracking, and synthesis approach are reported in Methods.
Referencing	2	Principal statements are supported with primary studies, reviews, and meta-analyses.
Scientific reasoning	2	Evidence level, eligibility restrictions, population source, extrapolation, and limitations are explicitly presented.
Presentation of relevant endpoint data	2	Tables 1, 2, and 5 report functional and geriatric outcomes and identify missing evidence.
Total	12/12	Author self-assessment.

The EQUATOR Network reporting-guideline library was consulted, but no dedicated reporting guideline for general narrative reviews was identified. SANRA was therefore used as the most directly applicable quality-assessment framework. SANRA is not a reporting guideline, and this author self-assessment does not imply methodological equivalence to a systematic review.
